# The Role of Satellite Glial Cells, Astrocytes, and Microglia in Oxaliplatin-Induced Neuropathic Pain

**DOI:** 10.3390/biomedicines8090324

**Published:** 2020-09-02

**Authors:** Ji Hwan Lee, Woojin Kim

**Affiliations:** Department of Physiology, College of Korean Medicine, Kyung Hee University, Seoul 02453, Korea; mibdna@khu.ac.kr

**Keywords:** astrocytes, chemotherapy-induced neuropathic pain, glia, microglia, oxaliplatin

## Abstract

Oxaliplatin is a third-generation platinum-based chemotherapeutic drug. Although its efficacy against colorectal cancer is well known, peripheral neuropathy that develops during and after infusion of the agents could decrease the quality of life of the patients. Various pathways have been reported to be the cause of the oxaliplatin-induced paresthesia and dysesthesia; however, its mechanism of action has not been fully understood yet. In recent years, researchers have investigated the function of glia in pain, and demonstrated that glia in the peripheral and central nervous system could play a critical role in the development and maintenance of neuropathic pain. These results suggest that targeting the glia may be an effective therapeutic option. In the past ten years, 20 more papers focused on the role of glia in oxaliplatin-induced thermal and mechanical hypersensitivity. However, to date no review has been written to summarize and discuss their results. Thus, in this study, by reviewing 23 studies that conducted in vivo experiments in rodents, the change of satellite glial cells, astrocytes, and microglia activation in the dorsal root ganglia, spinal cord, and the brain of oxaliplatin-induced neuropathic pain animals is discussed.

## 1. Introduction

Oxaliplatin is a platinum-based chemotherapeutic agent that is widely used to treat colorectal and other types of cancer [1]. Although it is widely used as it causes no nephrotoxicity or ototoxicity compared to the previous generation of platinum-based drugs [2], oxaliplatin can induce reversible peripheral neurotoxicity during or after the treatment in up to 90% of cases [3]. This neuropathy is characterized by symmetrical paresthesia and dysesthesia in the extremities, which is aggravated by cold exposure [4,5]. In cancer patients, this neuropathy can be hazardous, as it can interrupt the treatment schedule by necessitating dose reduction or discontinuation.

Since the development of the third-generation chemotherapeutic agent, experimental studies have reported various pathways of its mechanism. Ion channel dysfunction was suggested to be the cause of pain as the function of sodium and potassium channels in the dorsal root ganglia (DRG) neurons altered after oxaliplatin injection [6,7]. In addition, involvement of environmental changes, such as toxic effect of mitochondria [8] and pH acidification [9], in the DRG were reported to cause allodynia and hypersensitivity. Nevertheless, the underlying mechanism of action of oxaliplatin has not been fully understood yet.

In recent years, accumulating studies have revealed glia as an important contributor to pain [10]. Activation of glia was shown to increase after nerve injury, inflammation, and infection, and their activities were mostly related to development, maintenance, and potentiation of neuropathic pain [11]. Furthermore, injection of minocycline, which can inhibit microglia activation, prevented the development of neuropathic pain [12], whereas fluorocitrate administration, which is known to inhibit the activation of astrocytes, alleviated established neuropathic pain [13]. Altogether, these results suggest that glia may also play a critical role in the development and maintenance of oxaliplatin-induced neuropathic pain; however, relatively little is known about the function of glia in response to chemotherapy-induced peripheral neuropathy.

For several years, our lab has focused on trying to understand the underlying mechanism of oxaliplatin-induced pain and finding an effective method to attenuate the increased pain behavior caused by the agent infusion. By using an animal model of oxaliplatin-induced neuropathic pain [14,15], we have demonstrated that various drugs and treatment methods such as gabapentin [16], duloxetine [5], acupuncture [16], and bee venom [17] can attenuate cold and mechanical hypersensitivity induced by oxaliplatin. Moreover, we have shown that after oxaliplatin injection the activities of spinal astrocytes and microglia increased and that upregulated glial expression was lowered in the group where the pain was attenuated [18,19,20]. Thus, based on the data obtained from other studies and ours, the aim of this review was to understand the role of glia in the development and maintenance of oxaliplatin-induced neuropathic pain and to analyze whether targeting glia could be an effective therapeutic approach. Since the study published by Zheng et al. [21], more studies have investigated the relation of glial function and oxaliplatin-induced peripheral neuropathy in rodents. Thus, in this review, by analyzing all studies that conducted behavioral tests and glial observation in the peripheral and the central nervous system (satellite glial cells (SGCs), astrocytes, and microglia), we discuss the change of glial activities and their role in oxaliplatin-induced neuropathic pain (Table 1).

## 2. Results

### 2.1. Dorsal Root Ganglia (DRG)

#### Satellite Glial Cells (SGCs)

One week after oxaliplatin injection, Warwick et al. [22] demonstrated that the pain threshold decreased in rats, whereas the number of glial fibrillary acidic protein (GFAP) expressing SGCs surrounding DRG neurons by more than 50% of their circumferences increased compared to vehicle treated rats. Furthermore, coupling between SGCs surrounding different neurons increased five-fold one week after oxaliplatin injection. Intraperitoneal injection of carbenoxolone (CBX, 50 mg/kg), which blocked the gap junction of SGCs, increased the lowered paw withdrawal threshold when measured one hour after injection. In agreement with this finding, Mannelli et al. [23] demonstrated that the number of activated SGCs in the DRG significantly increased after oxaliplatin injection on D21, when thermal allodynia and mechanical hyperalgesia were significantly induced following oxaliplatin injection in rats. In another study conducted by Mannelli et al. [24] multiple oxaliplatin treatment induced increase of SGCs in the DRG. However, intrathecal injection of minocycline or fluorocitrate, which is a microglia and astrocytes inhibitor, respectively, significantly attenuated oxaliplatin-induced pain, although they failed to decrease the increased SGCs level in the DRG.

### 2.2. Spinal Cord

#### 2.2.1. Astrocytes

In the study conducted by Yoon et al. [25], glial fibrillary acidic protein (GFAP) expression was increased at D7, but not at D14 after oxaliplatin injection. Morphology of astrocytes also changed as cell bodies hypertrophied with thickened and elongated processes. However, these morphological modifications disappeared on D14. They also showed that astrocyte-specific gap junction protein connexin 43 (Cx43), which is the primary component of intracellular gap junction connections in astrocytes, was significantly increased in spinal dorsal horn after the chemotherapeutic agent administration both on D7 and D14. Co-immunofluorescent staining further confirmed that Cx43 was co-localized with GFAP in both spinal dorsal horn and white matter. However, the expression level of neuron specific gap junction protein (Cx32) and oligodendrocyte-specific gap junction protein (Cx36) remained unchanged. Furthermore, they showed that blocking the astrocyte gap junction with 25 μg of CBX (i.t., once a day for seven days) before the development of mechanical allodynia could prevent the inducement of pain, whereas, once the pain was established, CBX had no effect on oxaliplatin-induced allodynia. In the experiments conducted by Mannelli et al. [23], multiple oxaliplatin injections induced thermal and mechanical hypersensitivity from D7 to D21. Astrocytic density was significantly increased in the superficial laminae of the spinal dorsal horn after oxaliplatin injection until D21. The greater difference was shown on D7 when GFAP density was 54% higher in the oxaliplatin group than the vehicle treated rats. However, contrary to the report by Yoon et al. [25], the morphology of astrocytes did not changed in their study.

Moreover, Ahn et al. [18] showed that a single intraperitoneal injection of oxaliplatin (6 mg/kg) can induce mechanical and cold allodynia, and that GFAP expression can increase in the spinal cord. Astrocytes had thicker processes than the control. However, administration of gyejigachulbu-tang (GBT, 400 mg/kg, p.o.), a herbal formula consisting of seven different herbs, significantly attenuated pain and spinal astrocytes activation. In the study by Mannelli et al. [26], oxaliplatin induced an increase of astrocytes. However, acetylcholine receptor 7 agonist (α7nAChR) (R)-(−)-3-methoxy-1-oxa-2,7-diaza-7,10-ethanospiro[4.5]dec-2-ene sesquifumarate ((R)-ICH3) and PNU-282987, which were able to prevent oxaliplatin-induced cold and mechanical pain, increased astrocyte expression in the spinal cord. These agonists further increased the number of spinal astrocytes even in the absence of oxaliplatin. In another paper published by Mannelli et al. [24], the density of spinal GFAP-positive cells were increased on D7, D14, and D21 after oxaliplatin injection. This increase induced by oxaliplatin was prevented by co-administration of astrocyte inhibitor fluorocitrate (1 nmol/h). Fluorocitrate also prevented the development of thermal and mechanical hypersensitivity. Robinson et al. [27] showed that GFAP expression in the spinal cord increased after oxaliplatin treatment on D7 and D14. Pain was measured using the von Frey test and the threshold to mechanical stimuli significantly decreased on D14 compared to the control. Intraperitoneal injection of microglia inhibitor minocycline attenuated both the pain and GFAP upregulation. In accordance with other studies, Janes et al. [28] reported that multiple oxaliplatin injection upregulated GFAP-positive cells bilaterally within the superficial dorsal horn (laminae I and II) at D25. This increase was blocked by Gi/Gq-coupled A_3_ adenosine receptor (A3AR) agonist MRS5698 administration (0.1 mg/kg, i.p.). In their animal model, oxaliplatin administration induced mechanical allodynia and hyperalgesia from D11 to D25 and MRS5698 administration significantly blocked the pain.

In disagreement with other studies, Kim et al. [29] reported that oxaliplatin administration did not induce upregulation of GFAP-positive cells in the spinal cord. In their study, only microglia but not astrocytes were activated after oxaliplatin injection when measured on D28. However, they stated that GFAP cell density was slightly higher in the superficial laminae of oxaliplatin treated rats (although statistically non-significant). Mannelli et al. [30] showed that repeated treatment of oxaliplatin upregulated GFAP intensity in the superficial laminae of the spinal cord. Multiple N-palmitoylethanolamine (PEA, 30 mg/kg, i.p.) injection significantly decreased oxaliplatin-induced pain and lowered the increased spinal GFAP intensity. Deng et al. [31] demonstrated that intraperitoneal injection of oxaliplatin induced mechanical allodynia and hyperalgesia. GFAP-positive cells in the spinal dorsal horn also increased as well as the soma and the processes of astrocytes. This increase was attenuated by Wen–Luo–Tong (WLT) treatment; the paws and tails of animals were soaked in WLT (25–28 °C, 20 min, twice daily) from D1 to D30. Similar to the study by Ahn et al. [18], Kim et al. [19] demonstrated that a single injection of oxaliplatin can induce pain and spinal astrocytic upregulation. GFAP-positive cells were significantly increased in the oxaliplatin treated group compared to the vehicle treated group. However, GFAP-positive cells increased in the spinal cord and pain behavior was significantly attenuated by water extract of cinnamomi cortex (WECC, 200 mg/kg, p.o.) administration. Pacini et al. [32] demonstrated that oxaliplatin injection significantly increased the GFAP-positive cells in the spinal cord on D21. α-Conotoxin RgIA, a peptide that potently blocks the α9α10 nicotinic acetylcholine receptor (nAChR) subtype (2 and 10 nmol injected intramuscularly once a day concomitantly with oxaliplatin treatment), reduced the oxaliplatin-induced paw withdrawal threshold and licking latency to cold stimuli. It also prevented the increase in the number of GFAP-positive cells. Jung et al. [20] also showed that GFAP-positive cells increased after oxaliplatin administration and that oral administration of Buja (300 mg/kg) significantly decreased the upregulated GFAP-positive cell numbers. In the study by Makker et al. [33], although consecutive injection of oxaliplatin significantly induced reduction in paw thresholds on D8, D13, and D16 in C57BL/6J mice, the astrocytic number in the spinal dorsal horn remained unchanged. This may be due to the difference in the animal used compared to other studies (C57BL/6J mice versus Sprague–Dawley (SD) and Wistar rat).

In accordance with their previous studies, Mannelli et al. [34] showed that multiple injections of oxaliplatin could induce a decrease in the paw withdrawal threshold compared to the control on D21. Also, the number of GFAP-positive astrocytes increased as well as the body size. Treatment with 50% hydroalcoholic extracts of astragali radix (50% HA) reduced both the pain and the number of spinal astrocytes. In the study of Wang et al. [35], 5 mg/kg consecutive administration of oxaliplatin for four days induced paw withdrawal threshold and thermal hyperalgesia in rats. In the superficial dorsal horn, GFAP expression significantly increased compared to vehicle treated rats. The morphology of astrocytes changed as cells appeared hypertrophic with thicker processes. Single intraperitoneal melatonin injection (20 mg/kg, i.p.) on D7, significantly increased the paw withdrawal threshold and thermal hyperalgesia lowered by oxaliplatin. GFAP expression was also attenuated in the spinal cord. In the study by Areti et al. [36] in accordance with other studies, intraperitoneal injection of oxaliplatin induced the development of mechanical and thermal hyperalgesia. Moreover, in the L4-L6 spinal cord of oxaliplatin treated rats, GFAP expression was significantly increased compared to the vehicle treated group. However, co-administration of oxaliplatin with rosmarinic acid (RA, 25 and 50 mg/kg, p.o.) attenuated oxaliplatin-induced mechanical and thermal hyperalgesia along with spinal GFAP expression. Tonkin et al. [37] observed that oxaliplatin injection induced a significant increase in the response rate of hind paw to 0.4 g von Frey filament on D13 and D16 compared to vehicle injected rats. By using Western blot analysis, they demonstrated that the protein level of Cx43 significantly increased after oxaliplatin injection. In the experiments conducted by Wahlman et al. [38], consecutive injection of oxaliplatin-induced mechano-allodynia and mechano-hyperalgesia in both rats and mice. Also, the expression of GFAP was increased in the spinal dorsal horn. To further observe the role of adenosine kinase (ADK) in CNS, they used co-localization analysis. Their results show that although the percentage of ADK-positive astrocytes did not change, the cellular volume occupied by ADK doubled in the oxaliplatin treated group animals. This increase was observed in the astrocyte nucleus and cytoplasm in soma that expanded into processes. In the study by Hao et al. [39], 15 injections of oxaliplatin induced mechanical allodynia and thermal hypersensitivity, and it increased GFAP expression in the spinal cord. Repeated injections of Huachansu (HA, 2.5 mg/kg, i.p.) significantly decreased the GFAP expression as well as pain behaviors in rats.

#### 2.2.2. Microglia

To assess whether chemotherapeutic agents can affect the spinal microglia as in traumatic injuries, Zheng et al. [21] observed the spinal cord 35 days after the administration of oxaliplatin. Although oxaliplatin injection induced mechanical allodynia and hyperalgesia; microglia were unchanged in the spinal cord suggesting that microglia have no significant role in the oxaliplatin-induced neuropathic pain. However, in disagreement with this finding, Mannelli et al. [23] demonstrated that Iba1 (microglia marker) positive microglia cells were increased after multiple oxaliplatin injections when measured on D7. However, on D14 and D21, this level returned to control level. In their study, neuropathic pain induced by oxaliplatin was significant on D7, D14, and D21 compared to control, suggesting that microglia may contribute to development but not maintenance of neuropathic pain.

In an acute model of oxaliplatin-induced neuropathic pain, Ahn et al. [18] showed that single injection of oxaliplatin (6 mg/kg, i.p.) can induce OX-42 (microglia marker) expression increase in the spinal cord. Microglia also showed morphological modifications after oxaliplatin treatment such as hypertrophic amoeboid shape with short processes. Oral administration of GBT significantly decreased the increased expression of microglia. Consistence with their previous study, Mannelli et al. [26] showed that the increase of oxaliplatin-induced Iba1 positive cells was not observable in the spinal cord when measured on D21. Showing that microglia are not activated in the late phase of the pain. The other paper published by Mannelli et al. [24] also support their previous results, as Iba1 positive cells were upregulated on D7 but not on D14 and D21. This increased activation of microglia in the spinal cord was decreased by intracatheter infusion of microglia inhibitor minocycline. Minocycline injection also decreased the mechanical and thermal hyperalgesia induced by oxaliplatin.

In disagreement with other papers, Kim et al. [29] showed that oxaliplatin injection increased the Iba1 positive cells in the late phase (D28); however, in their study, this was the only time point where measurement was conducted. Furthermore, they showed that PC injection (300 mg/kg, p.o.) significantly decreased the upregulated microglia in the spinal cord. However, Kim et al. [19] showed that a single oxaliplatin injection (6 mg/kg, i.p.) increased the number of Iba1 positive cells in the spinal cord on D5. This increase was significantly lowered by WECC (200 mg/kg, p.o.) injection. Based on their results, Pacini et al. [32] assessed that oxaliplatin did not induce an increase of Iba1 positive cells in the spinal cord but only in the brain areas involved in pain pathways. Jung et al. [20] showed that Iba1 positive cells increased after oxaliplatin administration. Oral administration of Buja (300 mg/kg, p.o.) significantly attenuated the increased Iba1 positive cell numbers. In the study by Makker et al. [33], although consecutive injection of oxaliplatin significantly induced reduction in paw thresholds on D8, D13, and D16 in C57BL/6J mice, Iba1 positive cell numbers in the spinal dorsal horn remained unchanged. In their study, the number of astrocytes unchanged as well. Also, in another study published by Mannelli et al. [34], microglia in the spinal cord remained unaltered to oxaliplatin treatment. Experiments conducted by Hao et al. [39] showed that oxaliplatin injection induced upregulation of Iba1 expressing cells compared to vehicle in rats. However, huachansu administration which significantly attenuated pain behavior induced by oxaliplatin injection failed to decrease the increased Iba1 expression in the spinal cord. By using a transgenic mice, Saika et al. [40] showed that oxaliplatin induced mechanical allodynia in mice. However, inhibition of spinal Iba1 cells by clozapine N-oxide (CNO, i.p.) did not relieve the pain in both male and female CX3CR1-hM4Di mice.

### 2.3. Brain

#### 2.3.1. Astrocytes

In the study by Mannelli et al. [23], multiple oxaliplatin injection induced an increase of GFAP-positive cells in the brain regions involved in pain sensation, such as the thalamus, neostriatum, anterior cingulate cortex (ACC), somatosensory cortex 1 (S1), ventrolateral periaqueductal grey (VL-PAG), and nucleus raphe magnus (NRM). However, in dorsolateral periaqueductal grey (DL-PAG) and medial forebrain bundle (mfb), the number of astrocytes did not increase significantly compared to vehicle. In another study by Mannelli et al. [26], astrocytes in the thalamus were not activated, whereas GFAP expression was upregulated in the spinal cord, S1 and VL-PAG. Repeated administration of (R)-ICH3 and PNU-282987 which showed analgesic effect against pain, further increased the GFAP expression already upregulated after oxaliplatin injection in the brain. In their next study, Mannelli et al. [30] showed that repeated treatment of oxaliplatin upregulated GFAP intensity in the brain S1 area. Administration of PEA (30 mg/kg daily i.p. for 21 days, from the first oxaliplatin injection) significantly decreased this increased GFAP intensity as well as the cold and mechanical allodynia in rodents. Pacini et al. [32] showed that GFAP-positive cells were upregulated in various brain areas, such as cyngulate cortex area 1 (Cg1), cyngulate cortex area 2 (Cg2), primary motor cortex (M1), secondary motor cortex (M2), S1, secondary somatosensory cortex (S2), granular insular cortex (GI), ventral posterolateral thalamic nucleus (VPL), mfb, periaqueductal grey (PAG), and CA2/CA3 fields of hippocampus. Multiple injection of RgIA (10 nmol/100 μL) failed to decrease the number of GFAP-positive cells in all observed areas of the brain, whereas the equal dose of RgIA significantly prevented the pain development induced by oxaliplatin injection. In the study by Mannelli et al. [34] astrocytes in the brain areas (Cg, S1, M1, PAG, and mfb) increased significantly compared to vehicle treated rats and 50% HA reduced upregulated astrcytes in all observed brain areas.

#### 2.3.2. Microglia

Contrary to the changes observed in the spinal cord, in the study of Mannelli et al. [23], microglia were mostly activated on D14 and not on D7 in the brain areas. The Iba1 positive cells increased in all observed brain areas after oxaliplatin injection; thalamus, neostriatum, ACC, S1, DL-PAG, VL-PAG, NRM, and mfb. The authors suggested that the difference in the activation time between the spinal cord and the brain areas may be due to the delayed activation in the brain area compared to spinal cord. In the other study by Mannelli et al. [26] Iba1 expression in the thalamus, S1 and DL-PAG increased significantly compared to control, whereas spinal Iba1 expression did not change on D21. Interestingly, although both α7nAChR agonists PNU-282987 and (R)-ICH3 significantly reduced pain behaviors evoked by oxaliplatin, the increased expression of Iba1 in the brain remained unaltered after their administration. Mannelli et al. [30] showed that repeated treatment of oxaliplatin increased Iba1 intensity in the brain S1 area, but not in the spinal cord. Multiple injections of PEA significantly decreased this increased Iba1 intensity in the S1. In accordance with previously mentioned studies, Pacini et al. [32] also demonstrated that Iba1 expression increased in the brain areas but not in the spinal cord after oxaliplatin injection. The brain areas observed were Cg1, Cg2, M1, M2, S1, S2, GI, VPL, mfb, PAG, and CA2/CA3 of the hippocampus. However, RgIA (10 nmol/100 μL) injection which attenuated mechanical and thermal pain induced by oxaliplatin, failed to decrease the increased density of microglia in all assessed brain areas. In agreement with the previous studies, the spinal microglia remain unchanged, whereas microglia in the brain (Cg, S1, M1, PAG, and mfb) increased to oxaliplatin treatment, in the study of Mannelli et al. [34]. Fifty percent HA reduced upregulated Iba1 positive cells in all observed brain areas with the exception of S1, which remained increased.

## 3. Discussion

**Figure 1:**
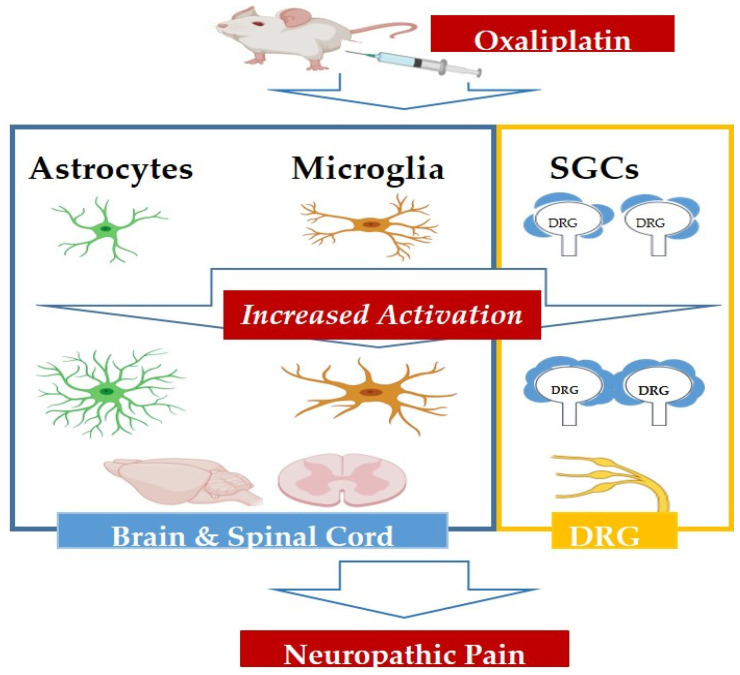
Schematic diagram of glial change in oxaliplatin-induced neuropathic pain. Administration of oxaliplatin induced astrocytes and microglia activation increase in the brain and spinal cord of rodents. The morphology of astrocytes and microglia changes to hypertrophic soma with thick processes and hypertrophic amoeboid shape soma with short processes, respectively. In the peripheral nervous system, SGCs in the DRG significantly increase their activity and coupling rate with SGCs surrounding neighboring neurons. These glial changes in the brain, spinal cord, and DRG may play an important role in the development and maintenance of oxaliplatin-induced neuropathic pain.

In this review, we observed the role of glia in oxaliplatin-induced neuropathic pain (Figure 1). Among 23 papers included, three studies assessed the change of SGCs [22,23,24], and the rest observed the alteration of astrocytes and microglia. In addition, the observed part also differed from paper to paper, as three studies focused on the DRG [22,23,24], whereas the rest analyzed the change in the spinal cord and the brain. Moreover, three studies observed the acute effect of oxaliplatin [18,19,20], whereas the rest assessed its chronic effect. In this review, we only included studies that conducted in vivo experiments along with glial assessment to better understand the change of glial activation in relation to different states of pain. Thus, in vitro studies with no pain assessments were excluded.

Oxaliplatin is known to have poor permeability across the blood–brain barrier (BBB) thus primarily affecting the DRG [41]. In normal conditions, the activities of SGCs are not significantly increased and only a small number of DRG neurons (4–9%) are known to share a common glial envelop [42], whereas when peripheral nerve damage-induced neuropathic pain occurs, the percentage of activated SGCs surrounding DRG neurons and coupling with other SGCs significantly increased [43,44]. In this review, three studies observed the change of SGCs activities in the DRG after oxaliplatin administration [22,23,24]. In accordance with other results, they showed that the number of activated SGCs significantly increased following oxaliplatin treatment, when the pain threshold was significantly lowered (increased pain) compared to control.

Regarding the spinal astrocytes, there is accumulating evidence that it plays a critical role in the formation and maintenance of neuropathic pain [45,46,47]. The number of astrocytes were reported to be increased in the spinal cord of trauma-induced neuropathic pain animal model [48]. In addition, astrocytes are known to modulate the excitatory neurotransmission in the CNS, as they encapsulate the synapses [49] and release various gliotransmitters [50]. In the studies included, oxaliplatin injection caused a significant increase of astrocytic density in the spinal cord. The increased activity of astrocytes was assessed by using GFAP labelling. In most of the studies included, the number of GFAP-positive cells in the spinal cord increased after single or multiple injection of oxaliplatin. However, two articles [29,33] reported that astrocytes were not altered following oxaliplatin injection. Kim et al. [29] demonstrated that the number of astrocytes were unchanged after multiple oxaliplatin administration (measured at D28). On the contrary, the activation of microglia was increased in their study. Makker et al. [33] also reported that astrocytes were not activated after oxaliplatin treatment at D13. Although it is hard to explain the discrepancies of the results, Makker et al. [33] suggested that contrary to other chemotherapeutic agents, such as paclitaxel, in oxaliplatin-induced neuropathic pain, astrocytes activation may be a contributing factor but not a critical phenotypic change for the development of pain in rodents. However, further studies may be needed to confirm this hypothesis. Furthermore, the difference in the experimental methods may also have affected the results as Kim et al. [29] used a relatively long period of oxaliplatin injection schedule (4 weeks) compared to others (single~3 weeks), and Markker et al. [33] used mice (C57BL/6J mice), whereas others used rats (SD and Wistar rats).

Similar to astrocytes, spinal microglia was also reported to play an important role in neuropathic pain [51,52]. However, unlike astrocytes, which are upregulated until the late phase of the nerve injury, microglia are mostly activated in the early phase of the pain [50]. In line with this, five studies [18,19,20,23,24] showed that the number of spinal Iba1 positive cells were significantly increased in the early phase of the pain (D5–D7). Mannelli et al. [23,24] further demonstrated that these upregulated microglia expression returned to the control level in the relative late phase of oxaliplatin-induced neuropathy (i.e., D14–D21). However, seven papers [21,26,28,30,32,33,34] reported that spinal microglia remained unaltered even after oxaliplatin injection. Considering the fact that they conducted the test in the late phase (D13–D35) of the pain, these results may also support the previous report that microglia are mostly activated in the early, but not late phase of the pain playing an important role in the development of neuropathic pain [53]. However, two papers demonstrated that microglial activation are increased in the late phase of oxaliplatin-induced pain (D21 and D28) [29,39]. By using the same injection schedule and dose of oxaliplatin as in the study by Manneli et al. [23], Hao et al. [39] showed that both astrocytes and microglia are activated in the spinal cord after oxaliplatin injection. However, Mannelli et al. reported that only spinal astrocytes, but not microglia are activated in the late phase of the pain (measured on D21). Moreover, Kim et al. [29], who reported that the number of spinal astrocytes remained unchanged after oxaliplatin injection, showed that the number of microglia increased, opposing the current hypothesis that spinal microglia contributes to the initial phase of the neuropathic pain, whereas astrocytes may be involved in its maintenance [54,55,56]. Nevertheless, some studies reported that microglia are involved not only in the initiation but also in ongoing maintenance of pain [12,57]. However, it should be noted that in the experiments conducted by Hao et al. [39] huachansu administration (2.5 mg/kg, i.p.), which significantly decreased oxaliplatin-induced hypersensitivity, failed to block the increased activation of spinal Iba1 positive cells, which suggests that microglia may have a limited role in the maintenance of oxaliplatin-induced neuropathic pain. Further well-designed studies are needed to draw any firm conclusions in the role of spinal astrocytes and microglia in oxaliplatin-induced neuropathic pain.

Brain glial activity is known to increase in pain [58,59]. In our study, five studies assessed the glial change in the brain after oxaliplatin injection [23,26,30,32,34]. They assessed the intensity of GFAP and Iba1 positive cells in all parts of the brain involved in pain transmission and descending inhibitory system, such as the thalamus, S1, and PAG. However, the activated phase of astrocytes and microglia were slightly different from that of the spinal glia. Less correlation with pain behavior was shown, as brain glia remained active even after mechanical and cold allodynia were attenuated in rats [26,32]. Furthermore, microglia activation that was not observable in the spinal cord was significantly increased in the brain areas [23,26,32]. Moreover, the peak activation of glia in the brain showed a little delay compared to spinal glia (D7 versus D14). It was suggested that this delayed activity may be due to the time needed for central sensitization [23].

In conclusion, in this review, we have discussed the change of glia activation in the DRG, spinal cord, and the brain after single or multiple administration of oxaliplatin. Although the number of studies is relatively small to draw a firm conclusion from, glia in the DRG and spinal cord were shown to be in close relation with pain behavior, whereas the association was shown to be lower in the brain areas. In the study by Mannelli et al. [24], intrathecal administration of minocycline and fluorocitrate, which could suppress the function of microglia and astrocytes respectively, significantly decreased the mechanical and thermal hypersensitivity induced by oxaliplatin. In other neuropathic pain models, injection of minocycline and fluorocitrate also successively reduced the pain [12,13,60,61]. Thus, based on these results, targeting the spinal glia to treat oxaliplatin-induced peripheral neuropathy may be an effective treatment approach. Future studies are needed to clarify which glia should be targeted in which phase of the pain to effectively decrease neuropathic pain. Moreover, the communications between astrocytes and microglia in oxaliplatin-induced neuropathic pain needs to be elucidated also.

## Figures and Tables

**Table 1:** Summary table of studies on glia and oxaliplatin-induced neuropathic pain.

Authors/ Years	Animal Type (Investigated Site)	Oxaliplatin Dosing Schedule/Cumulative Dose	Glial Type	Findings: Behavioral Assessments
				Findings: Glial Activation
Zheng et al. (2011) [21]	SD rat (spinal cord)	D0–4 (5 times)/10 mg/kg (i.p.)	Mic.	Oxaliplatin-induced mechanical hypersensitivity (von Frey test): D35; *p* < 0.05 (4 g); *p* < 0.01 (15 g) versus control
				No significant change in Iba1 expression after oxaliplatin treatment: D35; DH: 76.3 ± 4.2 (control) versus 67.9 ± 4.9; VH: 59.7 ± 5.3 (control) versus 55.3 ± 6.1
Warwick et al. (2012) [22]	Balb/c mice (DRG)	D0, D3 (twice)/8 mg/kg (i.p.)	SGCs	Oxaliplatin lowered the pain threshold by 70% (von Frey test): D7, *p* < 0.05 versus control Carbenoxolone (50 mg/kg, i.p.) increased the lowered pain threshold: *p* < 0.05 versus oxaliplatin
				Oxaliplatin increased the number of DRG neurons surrounded by SGCs (more than 50% of their circumference): 15% (control); 35% (oxaliplatin, *p* < 0.05 versus control) Carbenoxolone (i.p.) lowered the increased incidence of coupling rate after oxaliplatin injection: 5/60 (control), 23/56 (oxaliplatin, *p* < 0.05 versus control), 10/40 (50 mg/kg, NS versus oxaliplatin), 6/53 (100 mg/kg, *p* < 0.05 versus oxaliplatin)
Yoon et al. (2012) [25]	SD rat (spinal cord)	D1, D3, D5, D7 (4 times)/8 mg/kg (i.p.)	Ast.	Oxaliplatin decreased the pain threshold (von Frey test): D10, *p* < 0.05; D12 and D14, *p* < 0.01 versus control D0–7 carbenoxolone injection (25 μg, i.t.) prevented mechanical hypersensitivity D14–21 carbenoxolone injection (25 μg, i.t.) did not attenuate mechanical hypersensitivity
				Increased GFAP expression in the spinal cord after oxaliplatin injection: D7, *p* < 0.05 versus control (Hypertrophied cell bodies with thickened and elongated processes); D14, NS versus control Increased expression of Cx43 in the spinal dorsal horn: D7 and 14, *p* < 0.05 versus control D0–7 carbenoxolone injection (25 μg, i.t.) prevented the increase of GFAP expression
Mannelli et al. (2013) [23]	SD rat (brain, DRG, spinal cord)	D0–4, D7–11, D14–18 (15 times)/36 mg/kg (i.p.)	Ast. Mic. SGCs	Oxaliplatin induced cold hyperalgesia (cold plate test): 25.4 ± 0.9 s (control); 18.5 ± 0.8 s (D7, *p* < 0.05 versus control); 14 s (D14 and 21, *p* < 0.05 versus control) Oxaliplatin induced mechanical allodynia (von Frey test): 28.3 ± 1.2 g (control); 19.3 ± 1.3 g (D14, *p* < 0.05 versus control); 14.7 ± 1.5 g (D21, *p* < 0.05 versus control) Oxaliplatin induced mechanical hyperalgesia (paw-pressure test): 73.4 ± 2.1 g (control); 37.5 ± 2.8 g (D21, *p* < 0.05 versus control)
				The number of SGCs increased in the DRG: D21, 31.4 ± 5.4 (control); 92.1 ± 7.9 (oxaliplatin, *p* < 0.01 versus control) Iba1 cell density increased in the dorsal horn of the spinal cord: D7, 73% higher versus control, *p* < 0.05 (cell body increased, and branches of the processes shortened); D14, NS versus control; D21, NS versus control GFAP cell density in spinal superficial laminae increased after oxaliplatin injection: D7, D14, and D21, *p* < 0.05 versus control, highest on D7; 54% higher versus control. No altered morphology Iba1 positive cells increased after oxaliplatin injection: thalamus, neostriatum, S1, DL-PAG, NRM: D7, D14 and D21, *p* < 0.05 versus control; ACC and VL-PAG: D14, *p* < 0.05 versus control; mfb: D7 and D14, *p* < 0.05 versus control GFAP expressing cells increased after oxaliplatin injection: neostriatum, ACC, S1: D7, D14, and D21, *p* < 0.05 versus control; thalamus: D7 and D14, *p* < 0.05 versus control; VL-PAG: D21, *p* < 0.05 versus control; NRM: D14 and D21, *p* < 0.05 versus control; DL-PAG and mfb: NS versus control
Ahn et al. (2014) [18]	SD rat (spinal cord)	D0 (single)/6 mg/kg (i.p.)	Ast. Mic.	Oxaliplatin induced cold allodynia (TWL, 4 °C): D3 and D5, *p* < 0.001 versus control) Oxaliplatin induced mechanical hypersensitivity (von Frey test): D3, *p* < 0.05 versus control; D5, *p* < 0.001 versus control GBT (400 and 600 mg/kg, p.o.) attenuated the cold allodynia and mechanical hypersensitivity: D5, *p* < 0.001 and *p* < 0.01 versus oxaliplatin, respectively
				Oxaliplatin increased GFAP and OX-42 expression in the spinal cord: D5, 135.0 ± 13.5 (control); 216.3 ± 2.8 (oxaliplatin, *p* < 0.001 versus control); and 106.7 ± 11.9 (control); 204.0 ± 2.2 (oxaliplatin, *p* < 0.001 versus control) Hypertrophic with thick processes (astrocytes) and hypertrophic amoeboid shape with short processes (microglia) GBT (400 mg/kg, p.o.) suppressed the increase in the number of spinal GFAP and OX-42-positive cells: D5, *p* < 0.001 versus oxaliplatin
Mannelli et al. (2014) [26]	SD rat (brain, spinal cord)	D0–4, D7–11, D14–18 (15 times)/36 mg/kg (i.p.)	Ast. Mic.	Oxaliplatin induced cold hyperalgesia (cold plate test): 23.8 ± 1.2 s (control); 14.1 ± 0.8 s (D21, *p* < 0.01 versus control) Oxaliplatin induced mechanical allodynia (von Frey test): 21.0 ± 0.5 g (control); 13.8 ± 0.3 g (D21, *p* < 0.01 versus control) Oxaliplatin induced mechanical hyperalgesia (paw-pressure test): 68.3 ± 1.6 g (control); 41.7 ± 2.1 g (D21, *p* < 0.01 versus control) D0-20 α7 nAChR agonists (PNU-282987 and (*R*)-ICH3, 30 mg/kg, p.o.) injection prevented the pain behavior evoked by oxaliplatin (*p* < 0.01 versus oxaliplatin)
				No change in Iba1 positive cell number in the spinal cord after oxaliplatin treatment: D21 Iba1 expression increased in the brain after oxaliplatin: thalamus, S1, and DL-PAG (*p* < 0.05 versus control) PNU or (*R*)-ICH3 per se enhanced Iba1 cell density: in the thalamus by 60% and S1 by 108% (*p* < 0.05 versus control) Increase in GFAP-positive cell number in the spinal cord after oxaliplatin injection: D21 GFAP expression increased in the brain after oxaliplatin: S1 by 49% and VL-PAG by 50% (*p* < 0.05 versus control) PNU or (*R*)-ICH3 (30 mg/kg, p.o.) failed to reduce increased GFAP expression in the spinal cord PNU or (*R*)-ICH3 injection increased GFAP cell number in the thalamus and S1, but decreased in the VL-PAG (*p* < 0.05 versus oxaliplatin)
Mannelli et al. (2014) [24]	SD rat (spinal cord, DRG)	D0–4, D7–11, D14–18 (15 times)/36 mg/kg (i.p.)	Ast. Mic. SGCs	Oxaliplatin induced mechanical hyperalgesia (paw-pressure test): ± 70 g (control); 56.6 ± 1.9 g (D7, *p* < 0.05 versus control); 45.0 ± 4.1 g (D14, *p* < 0.01 versus control); 43.5 ± 1.9 g (D21, *p* < 0.01 versus control); Minocycline (12.5 nmol/h, i.t.) prevented mechanical hyperalgesia: by 50% (D7, *p* < 0.05); by 33% (D14, *p* < 0.05); by 60% (D21, *p* < 0.01); Fluorocitrate (1 nmol/h, i.t.) prevented hyperalgesia: by 100% (D7, *p* < 0.01); by 80% (D14, *p* < 0.01); 87% (D21, *p* < 0.01) Oxaliplatin induced mechanical allodynia (von Frey test): ± 28 g (control); 19.2 ± 0.6 g (D14, *p* < 0.05); 14.2 ± 0.5 g (D21, *p* < 0.01). Minocycline prevented by 60% (D14, *p* < 0.05); 90% (D21, *p* < 0.01); Fluorocitrate prevented by 93% (D14, *p* < 0.01); 94% (D21, *p* < 0.01). Oxaliplatin induced cold hyperalgesia (cold plate test): ± 23 s (control), 17.0 ± 1.5 g (D7, *p* < 0.05), 16.1 ± 0.8 g (D14, *p* < 0.01), and 12.4 ± 1.1 g (D21, *p* < 0.01); Minocycline prevented by 80% (D21, *p* < 0.01); Fluorocitrate prevented by 100% (D7, *p* < 0.01), by 90% (D14, *p* < 0.01), and by 95% (D21, *p* < 0.01)
				Oxaliplatin increased Iba1 positive cells in the spinal cord: D7, *p* < 0.01 versus control. Minocycline prevented this increased: D7, *p* < 0.01 versus oxaliplatin; D14, NS versus control; D21, NS versus control Oxaliplatin increased GFAP expressing cell number in the superficial laminae of the dorsal horn: D7, D14, and D21, *p* < 0.01 versus control. Fluorocitrate prevented this increase: D7, D14, and D21, *p* < 0.01 versus oxaliplatin Minocycline and fluorocitrate did not decrease the number of SGCs increased in DRG after oxaliplatin treatment: D21; 10.7 ± 1.3 (control); 23.5 ± 2.7 (oxaliplatin, *p* < 0.05 versus control); 20.4 ± 3.6 (minocycline, NS versus oxaliplatin); 22.7 ± 4.1(fluorocitrate, NS versus oxaliplatin)
Robinson et al. (2014) [27]	SD rat (spinal cord)	D1, D3, D5, D7 (4 times)/8 mg/kg (i.p.)	Ast.	Oxaliplatin induced mechanical allodynia (von Frey test): D14, 20.6 ± 1.8 g (control); 12.0 ± 1.4 g (oxaliplatin, *p* < 0.05 versus control); D0-8 treatment of minocycline (25 mg/kg, i.p.) prevented this change: D14
				Oxaliplatin increased GFAP fluorescence intensity in the spinal cord: D7, 131 ± 9.4% of control (*p* < 0.001); D14, 122 ± 4.7% of control (*p* < 0.001) D0–8 treatment of minocycline showed an increase of intensity at D7, 115 ± 13.5% of control (NS) and decrease at D14, 91 ± 20.2% of control (NS)
Janes et al. (2015) [28]	SD rat (spinal cord)	D0–4 (5 times)/10 mg/kg (i.p.)	Ast. Mic.	Oxaliplatin induced mechanical hypersensitivity (von Frey test): D11, D17, and D25 (*p* < 0.05 versus control) D0–4 A3AR agonists MRS5698 (0.1 mg/kg/day, i.p.) injection prevented the development of mechanical hypersensitivity: D11, D17, and D25 (*p* < 0.05 versus oxaliplatin)
				No change in spinal expression of OX42 after oxaliplatin treatment: D25 GFAP expression increased bilaterally within the superficial dorsal horn: *p* < 0.05 versus control Enhanced expression of GFAP was suppressed by treatment of MRS5698: *p* < 0.05 versus oxaliplatin
Kim et al. (2015) [29]	SD rat (spinal cord)	Twice a week for 4 weeks (8 times)/32 mg/kg (i.p.)	Ast. Mic.	Oxaliplatin induced mechanical allodynia (von Frey test): D21, *p* < 0.05; D28, *p* < 0.01 versus control Oxaliplatin induced thermal pain (hot plate test, tail-flick test): D14, *p* < 0.01 versus control; D21, *p* < 0.05 versus control; D28, *p* < 0.001 versus control PC (300 mg/kg, p.o.) treatment decrease mechanical allodynia induced by oxaliplatin: D28, *p* < 0.05 versus oxaliplatin Increased thermal pain reduced after PC treatment: D21, *p* < 0.05; D28, *p* < 0.01 versus oxaliplatin
				No change in GFAP expression in the spinal cord after oxaliplatin treatment: D28 PC administration decreased the number of Iba1 positive cells increased after oxaliplatin treatment in the spinal cord: D28, 10.00 ± 2.12 (control); 16.67 ± 4.27 (oxaliplatin, *p* < 0.01 versus control); 11.80 ± 3.11 (PC, *p* < 0.05 versus oxaliplatin)
Mannelli et al. (2015) [30]	SD rat (brain, spinal cord)	D0–4, D7–11, D14–18 (15 times)/36 mg/kg (i.p.)	Ast. Mic.	Oxaliplatin induced cold hyperalgesia (cold plate test): 21.3 ± 0.8 s (control); 11.5 ± 0.6 s (D21, *p* < 0.01 versus control) Single PEA (30 mg/kg, i.p.) injection relieved pain for 30–60 min after administration (*p* < 0.01 versus oxaliplatin) D0–20 PEA (30 mg/kg/day, i.p.) administration decreased mechanical allodynia about 40% (*p* < 0.05 versus oxaliplatin) Oxaliplatin induced mechanical allodynia (von Frey test): 32.1 ± 1.1 g (control), 21.6 ± 1.1 g (D21, *p* < 0.01 versus control) D0-20 PEA treatment prevented pain threshold alteration by 55% (*p* < 0.01 versus oxaliplatin) Oxaliplatin induced mechanical hyperalgesia (paw-pressure test): 69.2 ± 1.7 g (control), 40.5 ± 1.3 g (D21, *p* < 0.01 versus control); D0-20 PEA treatment, prevented mechanical hyperalgesia by 62% (*p* < 0.01 versus oxaliplatin)
				D0–20 PEA treatment decreased increased expression of GFAP-positive cells by 66% in the dorsal horn of the spinal cord: D21, *p* < 0.05 versus oxaliplatin. Iba1 expression was not changed after oxaliplatin In S1, both GFAP and Iba1 positive cell number increased after oxaliplatin: D21, *p* < 0.05 versus control D0-20 PEA treatment decreased GFAP and Iba1 positive cell number: D21, *p* < 0.05 versus oxaliplatin
Deng et al. (2016) [31]	Wistar rat (spinal cord)	Twice a week (not mentioned)/36 mg/kg (i.p.)	Ast.	WLT decrease oxaliplatin induced mechanical allodynia (von Frey test, 4 g): D31, 5.00 ± 5.35 (control); 46.25 ± 20.65 (oxaliplatin, *p* < 0.01 versus control); 16.25 ± 10.61 (WLT, *p* < 0.05 versus oxaliplatin) WLT decrease oxaliplatin induced mechanical hyperalgesia (von Frey test, 15g): D31, 18.75 ± 8.35 (control); 60.00 ± 16.04 (oxaliplatin, *p* < 0.01 versus control); 33.75 ± 15.06 (WLT, *p* < 0.01 versus oxaliplatin)
				GFAP-positive cell density change (IOD) after oxaliplatin and WLT treatment in the spinal dorsal horn: D31, 0.55 ± 0.07 (control); 1.27 ± 0.33 (oxaliplatin, *p* < 0.01 versus control); 0.61 ± 0.11 (WLT, *p* < 0.01 versus oxaliplatin) GFAP-positive cell density change (area μm^2^) after oxaliplatin and WLT treatment in the spinal dorsal horn: D31, 191.44 ± 171.04 (control); 1366.17 ± 486.86 (oxaliplatin, *p* < 0.01 versus control); 129.85 ± 54.31 (WLT, *p* < 0.01 versus oxaliplatin)
Kim et al. (2016) [19]	SD rat (spinal cord)	D0 (single)/6 mg/kg (i.p.)	Ast. Mic.	Oxaliplatin induced cold allodynia (TWL, 4 °C): D3–5, *p* < 0.001 versus control D0-4 WECC (200, 400 mg/kg, p.o.) administration showed potent analgesic effects: D3–5, *p* < 0.001 versus oxaliplatin Coumarin decreased oxaliplatin induced cold allodynia: D3 and 5, *p* < 0.001; D4, *p* < 0.01 versus oxaliplatin
				GFAP and Iba1 positive cells in spinal cord (laminae I–II) increased after oxaliplatin injection: *p* < 0.001 versus control D0–4 WECC (200 mg/kg, p.o.) administration suppressed the change in GFAP and Iba1 positive cells: GFAP (*p* < 0.001 versus oxaliplatin); Iba1 (*p* < 0.05 versus oxaliplatin)
Pacini et al. (2016) [32]	SD rat (brain, spinal cord)	D0–4, D7–11, D14–18 (15 times)/36 mg/kg (i.p.)	Ast. Mic.	Oxaliplatin induced mechanical hyperalgesia (paw-pressure test): ± 75 g (control); 60.1 ± 2.0 g (D7, *p* < 0.05 versus control); 47.4 ± 1.2 g (D14, *p* < 0.01 versus control); 44.6 ± 2.9 g (D21, *p* < 0.01 versus control); D0-21 RgIA (10 nmol/100 μL, i.m.) injection prevented pain development: D7, *p* < 0.05; D14 and D21; *p* < 0.01 versus oxaliplatin Oxaliplatin induced mechanical allodynia (von Frey test): ± 35 g (control); 20.2 ± 2.3 g (D14, *p* < 0.01 versus control); 15.0 ± 2.0 g (D21, *p* < 0.01 versus control); D0–21 RgIA injection increased the threshold (D14 and D21, *p* < 0.01 versus oxaliplatin) Oxaliplatin induced cold hyperalgesia (cold plate test): ± 23 s (control); 14.7 ± 0.9 g (D7, *p* < 0.01 versus control); 15.0 ± 0.6 g (D14, *p* < 0.01 versus control); 10.7 ± 1.6 g (D21, *p* < 0.01 versus control); D0–21 RgIA significantly delayed the latency (D7, *p* < 0.05 versus oxaliplatin; D14 and D21, *p* < 0.01 versus oxaliplatin group)
				No change in spinal expression of Iba1 after oxaliplatin treatment: D21, 40.7 ± 2.7 (control); 37.0 ± 4.4 (NS versus control) D0–21 RgIA (10 nmol) treatment prevented oxaliplatin-induced GFAP expression increase: 185.1 ± 21.3 (control); 303.0 ± 14.1 (*p* < 0.05 versus control); 201.3 ± 28.4 (*p* < 0.05 versus oxaliplatin) RgIA treated rats showed increase in the density of both Iba1 and GFAP expression in the brain: Cg1, Cg2, M1, M2, S1, S2, GI, VPL, mfb, PAG, CA2/CA3 (*p* < 0.05 versus control)
Jung et al. (2017) [20]	SD rat (spinal cord)	D0 (single)/6 mg/kg (i.p.)	Ast. Mic.	Oxaliplatin induced cold allodynia (TWL, 4 °C): D3, *p* < 0.05; D5, *p* < 0.001 versus control Oxaliplatin induced mechanical hypersensitivity (von Frey test): D3 and D5, *p* < 0.001 versus control D0-5 Buja (300 mg/kg, p.o.) administration prevented the development of cold allodynia (D5, *p* < 0.001 versus oxaliplatin) and mechanical hypersensitivity (D3 and D5, *p* < 0.001 versus oxaliplatin)
				GFAP and Iba1-positive cells increased in the spinal cord after oxaliplatin injection (*p* < 0.001 versus control) Buja only suppressed GFAP expressions (*p* < 0.001), but not Iba1 expression in cells increased by oxaliplatin
Makker et al. (2017) [33]	C57BL/6J mice (spinal cord)	D0, D2, D4, D6 (4 times)/20 mg/kg (i.p.)	Ast. Mic.	Oxaliplatin induced mechanical allodynia (von Frey test): D8, *p* < 0.01 versus control; D13, *p* < 0.001 versus control; and D16, *p* < 0.001 versus control
				No change of GFAP and Iba1 expression in the spinal cord after oxaliplatin injection: D13 Reduction in P2ry12 + homeostatic microglia in the spinal cord: *p* < 0.001 versus control
Mannelli et al. (2017) [34]	SD rat (BrainSpinal cord)	D0–4, D7–11, D14–18 (15 times)/36 mg/kg (i.p.)	Ast. Mic.	Oxaliplatin induced cold hyperalgesia (cold plate test): 24.1 ± 1.2 s (control); 16.2 ± 0.8 s (oxaliplatin, *p* < 0.05) Oxaliplatin induced mechanical allodynia (von Frey test): 23.7 ± 1.2 g (control); 13.8 ± 0.9 g (oxaliplatin, *p* < 0.05) Oxaliplatin induced mechanical hyperalgesia (paw-pressure test): 66.5 ± 1.9 g (control); 40.7 ± 1.8 g (oxaliplatin, *p* < 0.05 versus control) D0–21 treatment of 50% HA (300 mg/kg, p.o.) attenuated oxaliplatin-induced pain: D21, *p* < 0.01 versus oxaliplatin
				Increased number of GFAP but not Iba1 positive cells in the spinal cord after oxaliplatin injection: D21, *p* < 0.05 versus control; 50% HA reduced the number of GFAP-positive cells: *p* < 0.05 versus oxaliplatin Oxaliplatin increased both GFAP and Iba1 positive cells in the brain: Cg, S1, M1, PAG, and mfb (*p* < 0.05 versus control) D0–21 treatment of 50% HA (300 mg/kg, p.o.) reduced the number of the GFAP and Iba1 positive cells except in Cg (Iba1): *p* < 0.05 versus oxaliplatin
Wang et al. (2017) [35]	SD rat (spinal cord)	D0–3 (4 times)/20 mg/kg (i.p.)	Ast.	Oxaliplatin induced mechanical allodynia (von Frey test): D7, *p* < 0.05 versus control Oxaliplatin induced thermal pain (hot plate test, tail-flick test): D7, *p* < 0.05 versus control Single melatonin (20 mg/kg, i.p.) injection alleviated pain: D1 and D3, *p* < 0.05 versus oxaliplatin
				Enhanced GFAP expression after oxaliplatin treatment: *p* < 0.05 versus control; hypertrophic with thicker processes Melatonin (20 mg/kg, i.p.) decreased oxaliplatin-induced upregulation of GFAP expressions: *p* < 0.05 versus oxaliplatin
Areti et al. (2018) [36]	SD rat (spinal cord)	Twice a week for 4 weeks (8 times)/32 mg/kg (i.p.)	Ast.	Oxaliplatin induced cold allodynia (acetone spray test): D14–28, *p* < 0.001 versus control Oxaliplatin induced thermal hyperalgesia (hot/cold plate test): D14–28, *p* < 0.001 versus control Oxaliplatin induced mechanical hypersensitivity (von Frey test): D14–28, *p* < 0.001 versus control D0-28 RA (25 and 50 mg/kg, i.p.) treatment attenuated pain: D21–28, *p* < 0.001 versus oxaliplatin
				Oxaliplatin increased the GFAP expression in the L4-L6 spinal cord: *p* < 0.001 versus control D0-28 RA (25 and 50 mg/kg, i.p.) treatment significantly attenuated this increase: *p* < 0.01 versus oxaliplatin group
Tonkin et al. (2018) [37]	C57BL/6J mice (spinal cord)	D0, D2, D4, D6 (4 times)/20 mg/kg (i.p.)	Ast.	Oxaliplatin induced mechanical allodynia (von Frey test, 4g): D13, 2.1 folds; D16, 1.5 folds (*p* < 0.05 versus control) Cx43 mimetic (Peptide 5, 20 μM) injection had no effect on response rate: *p* > 0.05 versus control
				A significant increase in Cx43 protein levels in oxaliplatin treated mice:D13, 2.2 folds, *p* < 0.05 versus control
Wahlman et al. (2018) [38]	SD rat & C57BL/6J mice	D0–4 (5 times)/10 mg/kg (rats, i.p.), D0–4, D10–14 (10 times)/30 mg/kg (mice, i.p.)	Ast.	Oxaliplatin induced mechanical allodynia (von Frey test): D11, D17, and D25, *p* < 0.05 versus control Oxaliplatin induced mechanical hyperalgesia (paw pressure test): D11, D17, and D25, *p* < 0.05 versus control ABT-702 (30 nmol, i.t.) injection prevented these pain signs, but A3AR antagonist injection (MRS1523, 1 nmol, i.t.) blocked the effect: D11, D17, and D25, *p* < 0.05 versus oxaliplatin
				Increased GFAP expression after oxaliplatin treatment in the spinal dorsal horn: D25, *p* < 0.05 versus control Increased ADK expression in spinal dorsal horn after oxaliplatin injection: D11 and D25, *p* < 0.05 versus control No change in the percentage of ADK+ GFAP (co-localization) expression in the spinal cord: 44.49% ± 5.72 (control); 44.97% ± 12.23 (oxaliplatin, *p* = 0.95 versus control); but the cellular volume of astrocytes occupied by ADK (ADK+ voxels/GFAP+ voxels) increased: 0.20 ± 0.09 (control); 0.42 ± 0.08 (*p* < 0.05 versus control) Increased ADK signal was found in the astrocyte nucleus and cytoplasm in somas that expanded into processes
Hao et al. (2019) [39]	SD rat (spinal cord)	D0–4, D7–11, D14–18 (15 times)/36 mg/kg (i.p.)	Ast. Mic.	Oxaliplatin induced cold allodynia (acetone spray test): D7–21, *p* < 0.01 versus control Oxaliplatin induced thermal hyperalgesia (hot plate test): D7–21, *p* < 0.01 versus control Oxaliplatin induced mechanical allodynia (von Frey test): D7–21, *p* < 0.01 versus control Single Huachansu (2.5 g/kg, i.p.) injection alleviated cold allodynia (D21, *p* < 0.01 versus oxaliplatin), mechanical allodynia (D7, D14, D21, *p* < 0.01 versus oxaliplatin), and thermal hyperalgesia (D7, D14, D21, *p* < 0.01 versus oxaliplatin) D0–19 Huachansu (2.5 g/kg, i.p.) treatment alleviated cold allodynia (D21, *p* < 0.01 versus oxaliplatin), mechanical allodynia (D21, *p* < 0.01 versus oxaliplatin), and thermal hyperalgesia (D21, *p* < 0.01 versus oxaliplatin)
				Increased GFAP expression in the spinal cord after oxaliplatin injection: D21, *p* < 0.001 versus control Increased Iba1 expression in the spinal cord after oxaliplatin injection: D21, *p* < 0.001 versus control D0-19 Huachansu (2.5 g/kg, i.p.) treatment decreased upregulated GFAP expression: *p* < 0.01 versus oxaliplatin Huachansu failed to block the activation of Iba1 positive cells: *p* > 0.01 versus oxaliplatin
Saika et al. (2019) [40]	Transgenic mice (spinal cord)	D0, D2, D4, D6 (4 times)/20 mg/kg (i.p.)	Mic.	Oxaliplatin-induced mechanical allodynia (von Frey test) Single CNO (10 mg/kg, i.p.) injection did not prevent pain development in male and female CX3CR1-hM4Di mice
				HA-hM4Di was highly expressed in the SDH of CX3CR1-hM4Di mice In CX3CR1-hM4Di mice, HA-hM4Di overlapped with Iba1

**Abbreviations:** A3AR: A3 adenosine receptor, ABT-702: ABT 702 hydrochloride; 5-(3-Bromophenyl)-7-[6-(4-morpholinyl)-3-pyrido[2,3-d]byrimidin-4-amine hydrochloride, ACC: anterior cingulate cortex, ADK: adenosine kinase, Ast.: astrocytes, CA2: *cornu ammonis* 2 of hippocampus, CA3: *cornu ammonis* 3 of hippocampus, CNO: clozapine N-oxide, Cg1: cyngulate cortex area 1, Cg2: cyngulate cortex area 2, CX3CR1-hM4Di: CX3C chemokine receptor 1-human Gi coupled M4 muscarinic receptors, Cx43: connexin43, DL-PAG: dorsolateral periaqueductal grey, DH: dorsal horn, DRG: dorsal root ganglion, GBT: Gyejigachulbu-tang, GFAP: glial fibrillary acidic protein, GI: granular insular cortex, HA: 50% hydroalcoholic 50% extracts of Astragali radix, HA-hM4Di: Hemagglutinin A-human Gi coupled M4 muscarinic receptors Iba1: Ionized calcium binding adaptor molecule 1, IOD: integral optical density, i.p.: intraperitoneal, LPS: lipopolysaccharide, M1: primary motorcortex, M2: secondary motor cortex, Mic.: microglia, mfb: medial forebrain bundle, α7 nAChR: α7 nicotinic acetylcholine receptor, NRM: nucleus raphe magnus, mfb: medial forebrain bundle, NS: non-significant, OX-42: oxycocin-42, p2ry12: purinergic receptor P2Y12, PAG: periaqueductal grey, PC: phosphatidylcholine, PEA: N-Palmitoylethanolamine, (*R*)-ICH3: (*R*)-(−)-3-methoxy-1-oxa-2,7-diaza-7,10-ethanospiro[4.5]dec-2-ene sesquifumarate, RA: Rosmarinic acid, S1: primary somatosensory cortex, S2: secondary somatosensory cortex, SD rat: Sprague–Dawley rat, SGCs: satellite glial cells, TWL: tail withdrawal latency, VH: ventral horn, VL-PAG: ventrolateral periaqueductal grey, VPL: ventral posterolateral thalamic nucleus, WECC: water extract of cinnamomi cortex, WLT: Wen-luo-tong.
